# Supplementary material for: Foot-and-Mouth Disease Virus 3A Hijacks Sar1 and Sec12 for ER Remodeling in a COPII-Independent Manner
Source: Viruses. 2022 Apr 18;14(4):839. doi: 10.3390/v14040839 (PMC9028839; doi:10.3390/v14040839)
Supplement: Supplementary file 1 [file viruses-14-00839-s001.zip › viruses-1649680-new suppl.pdf]

Supplementary Information

Foot-and-mouth disease virus 3A hijacks Sar1 and Sec12 for ER remodeling in a COPII-independent manner

Heng-Wei Lee[1], Yi-Fan Jiang[1,2], Hui-Wen Chang[1,2], Ivan-Chen Cheng[1*] *

[1] *School of Veterinary Medicine, National Taiwan University, Taipei, 106, Taiwan*

[2] *Graduate Institute of Molecular and Comparative Pathobiology, School of Veterinary Medicine, National Taiwan University, Taipei, 106, Taiwan*

Correspondence: ivancheng@ntu.edu.tw (I.-V.C)

Supplementary Figure S1-S6

Supplementary Table S1

```
                                              Alpha-helix1   Alpha-helix2                                Hydrophobic region
                               20                   40                   60                   80
                                |                    |                    |                    |
O1Ca       ISIPSQKSVL YFLIEKGQHE AAIEFFEGMV HDSIKEELRP LIQQTSFVKR AFKRLKENFE IVALCLTLLA NIVIMIRETR 80
O/TAW/99   .......A.. .......... .......... .......... .......... .......... .......... .......... 80
O/TAW/97   .......... .......... .......... .......... .......... .......... V......... .....L.QA. 80
Consensus  ISIPSQK*VL YFLIEKGQHE AAIEFFEGMV HDSIKEELRP LIQQTSFVKR AFKRLKENFE *VALCLTLLA NIVIM*R**R

                              100                  120                  140
                                |                    |                    |
O1Ca       KRQKMVDDAV NEYIEKANIT TDDKTLDEAE KSPLETSGAS TVGFRERTLP GQKACDDVNS EPAQPVEEQP QAE 153
O/TAW/99   ...Q...... ........S.. .......... .N.......T ......K... .H..S..... ...K...... ... 153
O/TAW/97   ..YQS...PL D--------- -G.V..GD.. .N........ A......SPT E.GTRE.A.A ..VVFGR... R.. 143
Consensus  KR***VDD** *EYIEKA*IT T*D*TL**AE K*PLETSGA* *VGFRE**** ******D*N* EP*****EQP *AE
```

Figure S1. Alignment of the amino acid sequence of 3A among O1Campos (O1Ca, NCBI reference sequence CAC86575), O/TAW/99, and O/TAW/97 virus strains. The red boxes represent alpha-helix1 and alpha-helix2, while the yellow box indicates the hydrophobic region.

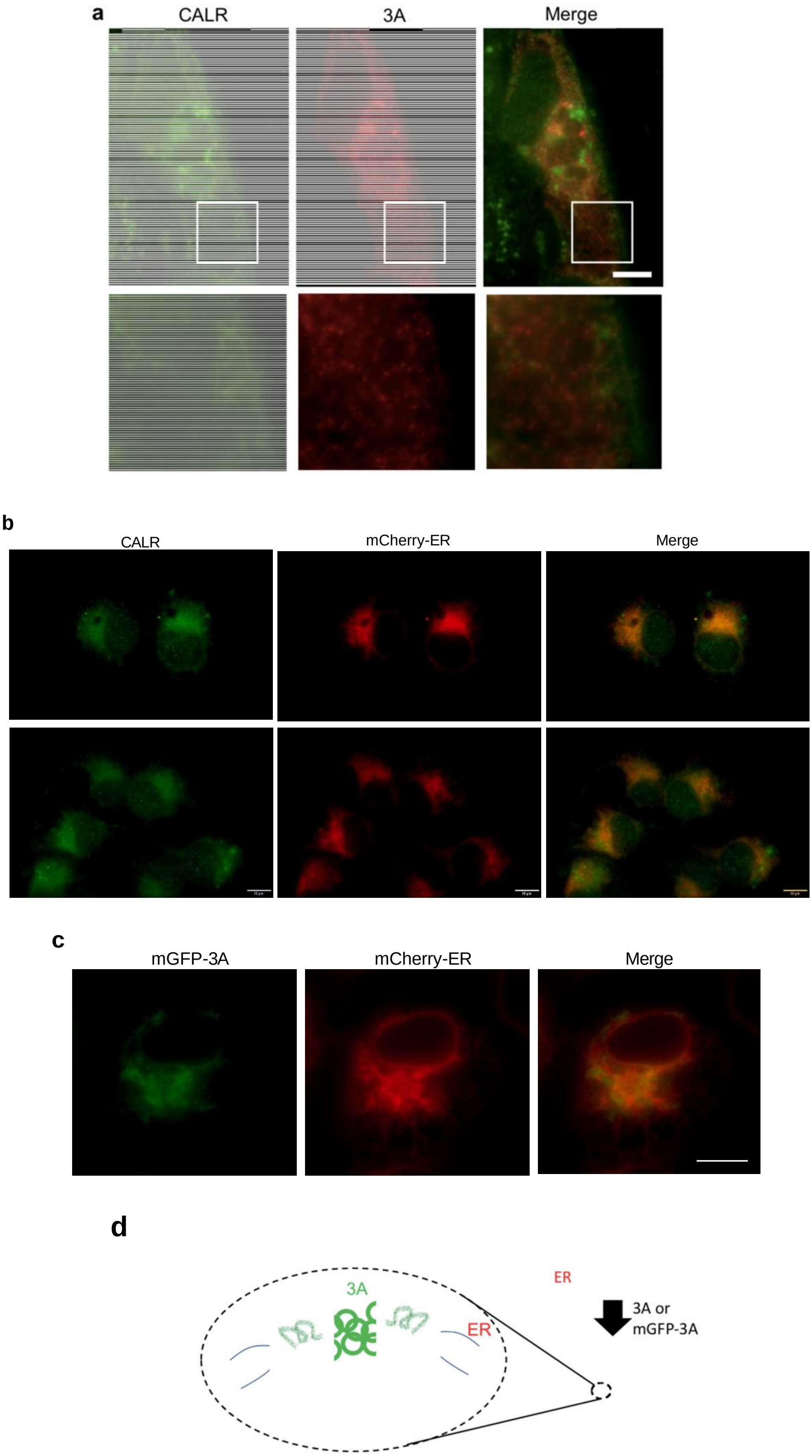
a
CALR
3A
Merge
b
CALR
mCherry-ER
Merge
c
mGFP-3A
mCherry-ER
Merge
d
3A
ER
ER
3A or
mGFP-3A

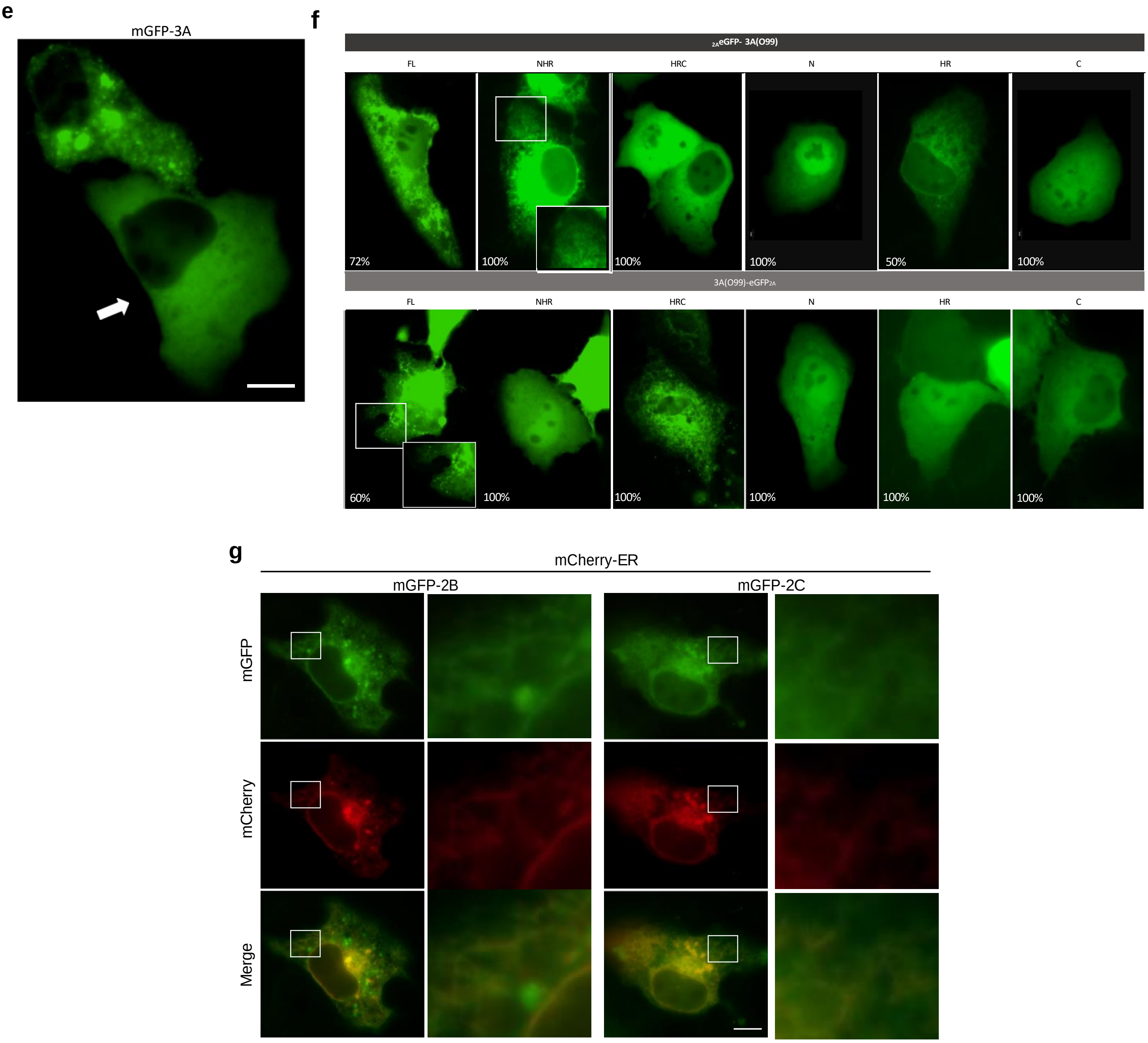

Figure S2. (**a**) Double immunofluorescence to 3A (red) and calreticulin (green) revealed with QA2 MAb and anti-calreticulin (CALR) rabbit antibody, followed by appropriate secondary antibodies (Alexa 594 conjugated anti-mouse antibody and FITC conjugated anti-rabbit antibody). (**b**) PK-15 cells expressing mCherry-ER were fixed and stained with anti-CALR rabbit antibody, followed by anti-rabbit antibody coupled with FITC. (**c**) After 3 hr post-transfection, PK-15 cells co-expressing mGFP-3A and mCherry-ER were examined by fluorescence microscopy. (**d**) The diagram depicts the modification of ER by 3A and the limitation of fluorescence microscopy. (**e**) PK-15 cells expressing mGFP-3A showed two different groups, diffuse-type (white arrow) and cells with multiple punctae. (**f**) The images from variant truncated 3A fused to eGFP. The N- or C-terminal eGFP was also fused to the 2A peptide of FMDV, which exhibited self-cleavage activity. It was originally designed for a cistronic co-expression system; however, we found that, if other protein genes that served 2A as linkage were added, the cleavage was not complete (data not shown).

Therefore, these plasmids were not used for further construction. However, mGFP-3A and $_{2A}$eGFP-3A truncated versions showed identical results; we thought 2A peptide would not interfere with the localization of 3A. Similar to mGFP-3A, 72% and 60% of $_{2A}$eGFP-3A and 3A- eGFP$_{2A}$ showed punctate patterns, respectively, while the others were diffuse-type. In addition, $_{2A}$eGFP-NHR and HRC- eGFP$_{2A}$ both showed clear reticular patterns. Only 50% of $_{2A}$eGFP-HR expressing cells appeared as reticular pattern; the others were diffuse. The other truncations distributed diffusely in the cytoplasm and nucleus. (**g**) PK-15 cells co-expressing mCherry-ER with mGFP-2B or mGFP-2C were examined for colocalization test. Scale bar, 10 μm.

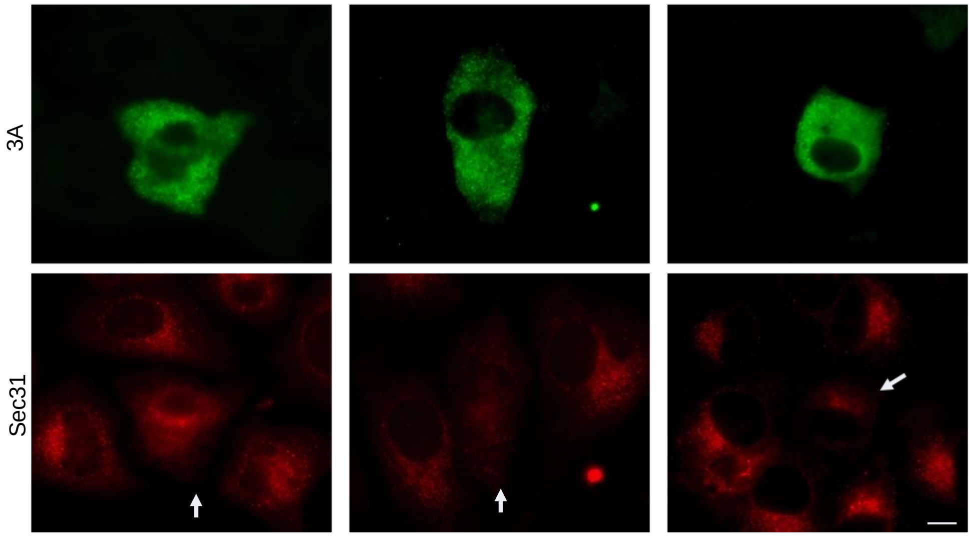

Figure S3. The transfected cells were stained with anti-Sec31A rabbit antibodies, followed by anti-rabbit secondary antibodies (red). Finally, 3A was identified with QA2-Dylight488 (green). The Sec31A signal in 3A-expressing cells (white arrows) was dispersed within the cytoplasm compared to non-transfected cells. Scale bar, 10 μm.

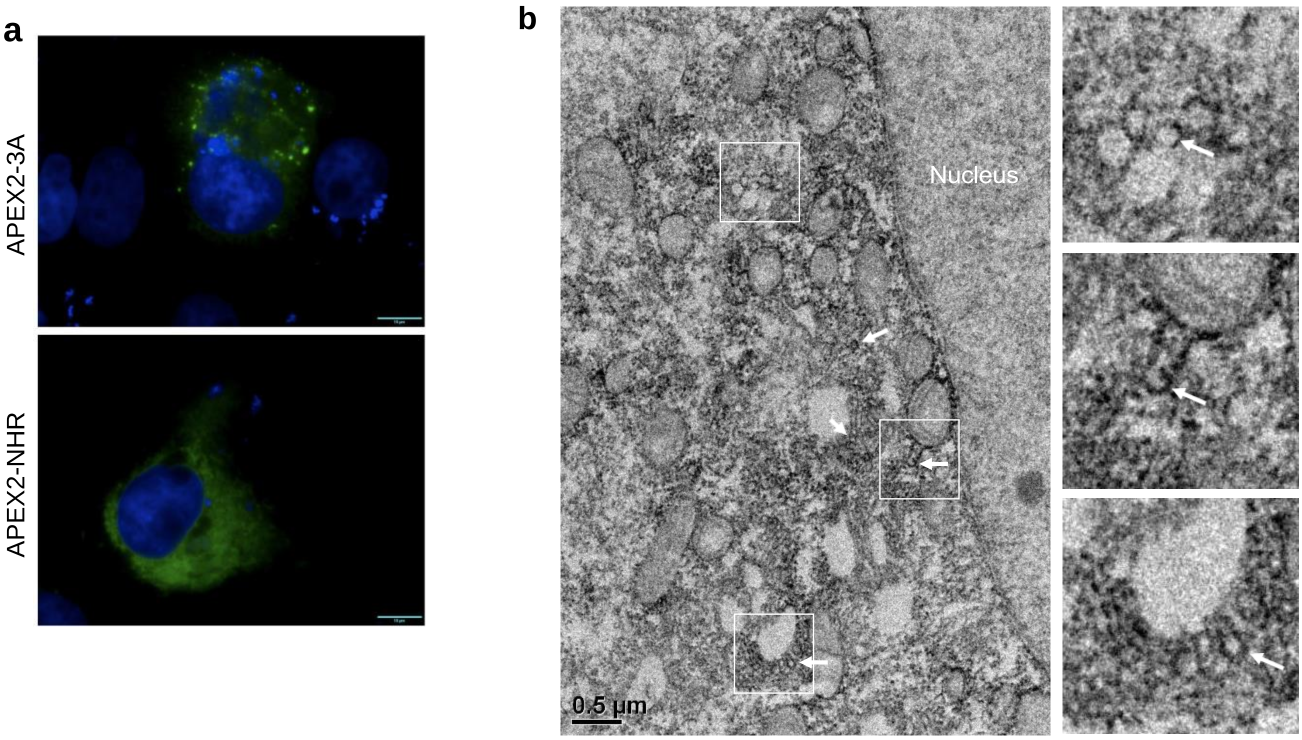

Figure S4. (**a**) The patterns of APEX2-3A and APEX2-NHR were examined under IFA by QA2 MAb. (**b**) The PK-15 cells' ultrastructure for expression of APEX2-N2HRC in TEM. The white arrows indicate modified structures around DAB reaction products.

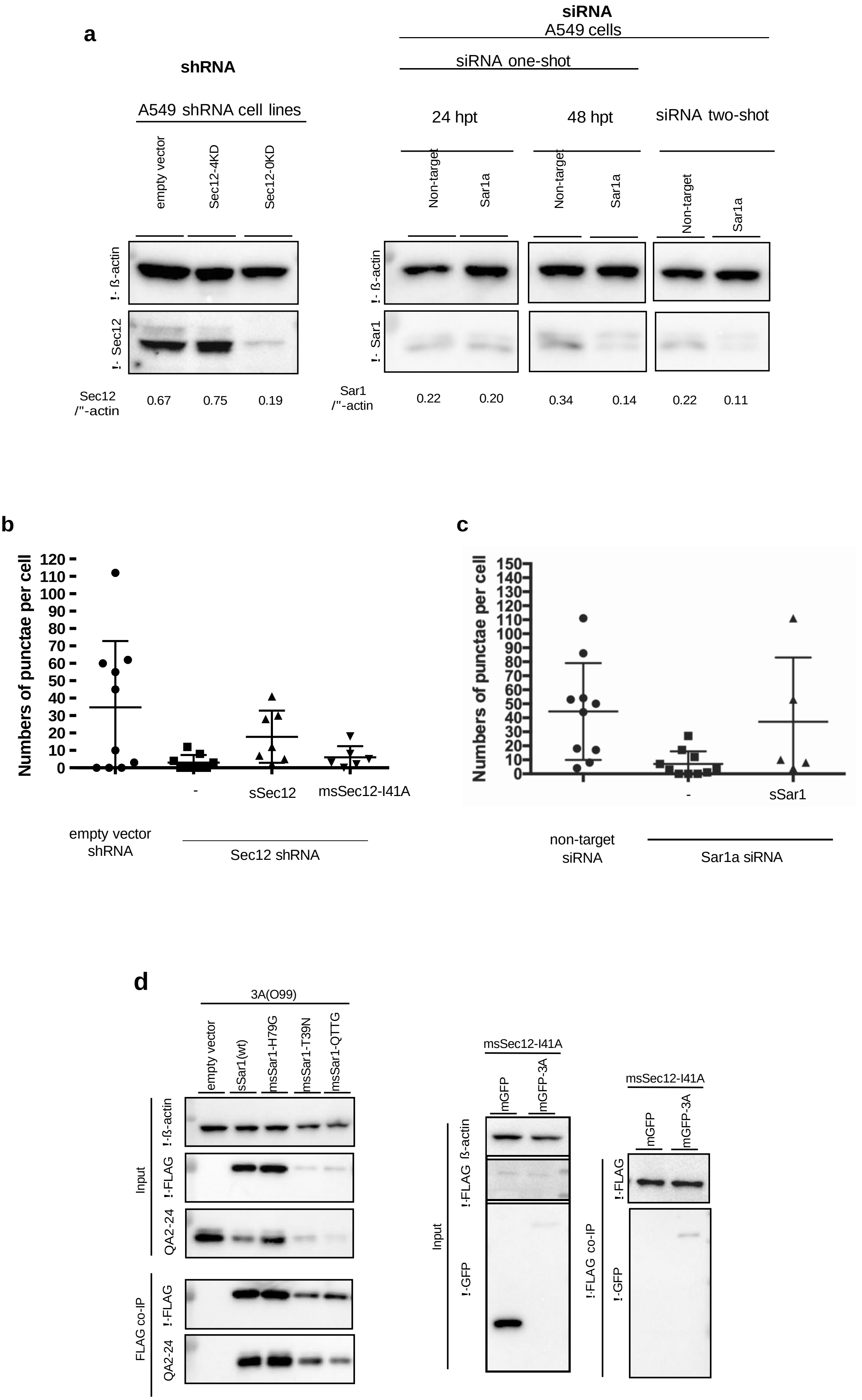

Figure S5. (**a**) For Sec12 knockdown, A549 cells were transfected with pLAS2w.Ppuro (empty vector), Sec12-4 (target sequence: GTGTGCTTCAACCACGATAAT) or Sec12-0 (target

sequence: GCTGGCCTAAAGATGCAATAA), followed by puromycin selection for more than 2

weeks. The cell lysates were examined by western blotting. The Sec12 knockdown cell line was successfully established for Sec12-0 and named A0 cells. For Sar1 knockdown, due to failure to establish the shRNA cell line, siRNA (target sequence: CCAGTTCCTAGGACTCTACAA) was chosen as an alternative. After siRNA transfection for 24 hr or 48 hr, cell lysates were examined by western blotting. To elevate knockdown efficiency, we performed double transfection at a 24 hr interval. Cells were harvested at the next 24 hours (after the last transfection of siRNA), which was adopted for the following experiments. The band intensities for indicated proteins were quantified in ImageJ software and standardized by β-actin. (**b, c**) The numbers of punctae were quantified in different condition for knockdown and re-expression assay. (**d**) HTK cell lysates, co-expressing 3A and wild-type (or mutants) of sSar1, were applied to co-immunoprecipitation assay. All mutants of sSar1 preserved the ability to interact with 3A. Empty vector: pcDNA-3.1(+). Similarly, in PK-15 cell lysates, the I41A mutation of Sec12 would not abrogate the interaction with 3A, as proved by mGFP-3A.

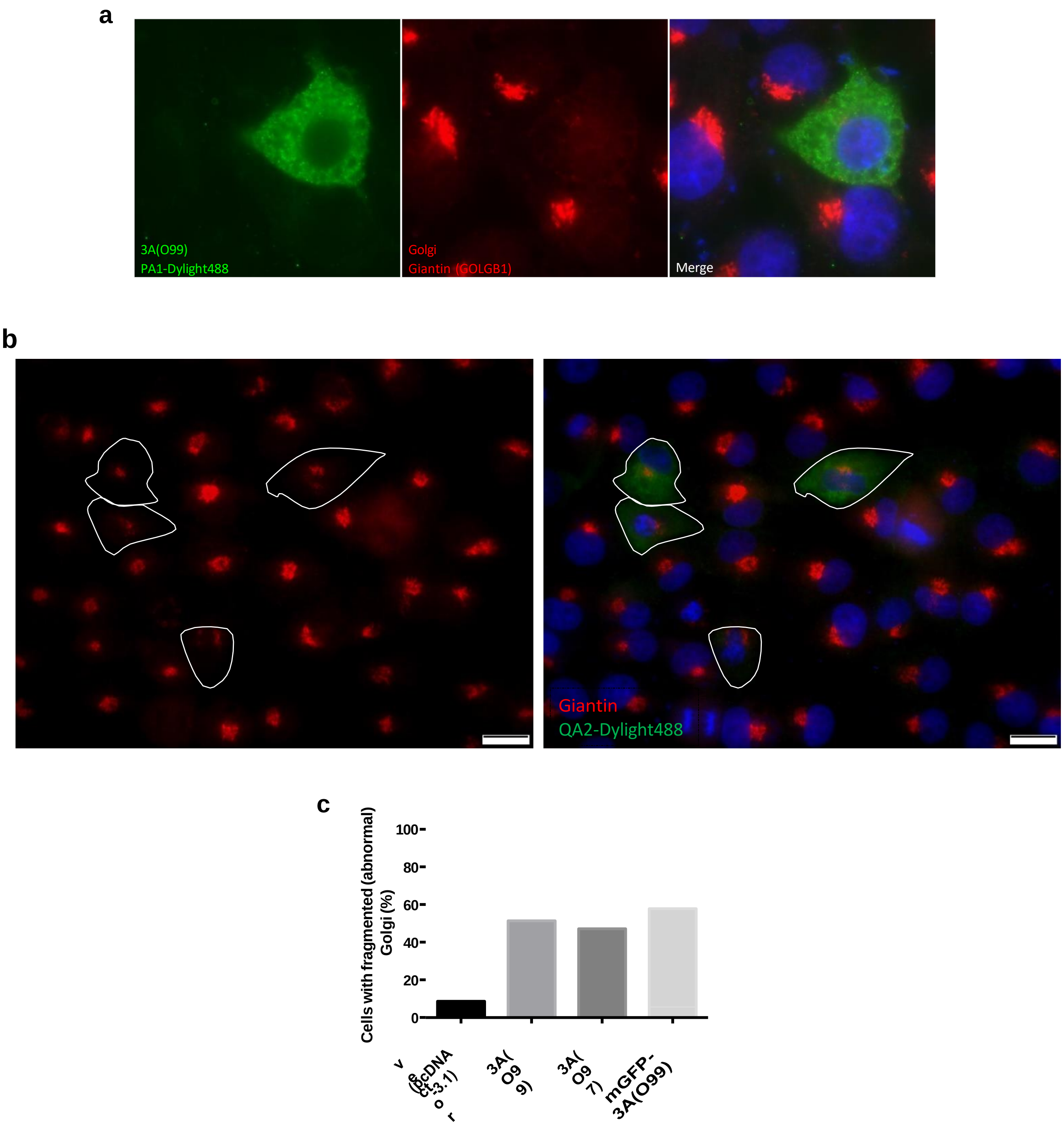

Figure S6. FMDV 3A expression resulted in the dispersal of the Golgi, shown by IFA. (**a**, **b**) The fixed PK-15 expressing 3A(O99) were subsequently incubated for anti-giantin rabbit antibodies, anti-rabbit antibodies-Alexa Fluor 594, and PA1-Dylight488 or QA2-Dylight488. Chromosomes were stained with Hoechst 33258. (**c**) PK-15 cells were transfected for 3A(O99), 3A(O97), and mGFP-3A(O99). Golgi integrity was examined in about 100 cells for each group, with mitotic cells excluded.

Supplementary Table S1. A list of cloning primers

Primer name	Sequence (5' to 3')
(mGFP-, eGFP- or GST-) HindIII-3A-XhoI (or XbaI)	
HindIII-3A(O99)-F	AAAAAGCTTGCCACCATGGCCATCTCAATTCCTTCCCAAAAGG
XhoI-3A(O99)His-R	AACTCGAGTCAATGATGGTGGTGGTGGTGTTCAGCTTGTGGTTGTTCTTC
HindIII-3A(O97)-F	AAAAAGCTTGCCACCATGGCCATTTCAATCCCTTCCCAGAAG
XhoI-3A(O97)His-R	AACTCGAGTCAATGATGGTGGTGGTGGTGTTCAGCTCGCGGTTGTTC
XhoI-3A(O99)-R	AACTCGAGTCATTCAGCTTGTGGTTGTTCTTC
HindIII-3A(O97)C-F	AAAAAGCTTGCCACCATGGCCCGCCAAGCGCGCAAG
XbaI-3A(O99)N-R	AATCTAGATTAGTTTTCCTTCAGGCGC
HindIII-3A(O99)HR-F	AAAAAGCTTATGGCCTTTGAGATAGTTGCCCTGTG
XhoI-3A(O99)HR-R	AAACTCGAGTCAGATCATGATCACTATGTTTGCC
HindIII-3A(O99)C-F	AAAAAGCTTATGGCCCGCGAGACTCGCAAG
HindIII-3A(O99)a2-F	AAAAAGCTTGCCACCATGGCCATCCAGCAGACCTCATTTG
XbaI-3A(O99)a1-R	TTTCTAGATTAGAGAGGCCGGAGCTCC
(APEX2) XhoI-3A-XbaI	
XhoI-3A(O99)-F	AACTCGAGATGGCCATCTCAATTCCTTCCCAAAAGG
XbaI-3A(O99)-R	AATCTAGATTATTCAGCTTGTGGTTGTTCTTC
XbaI-3A(O99)HR-R	AATCTAGATTAGATCATGATCACTATGTTTGCC
XhoI-3A(O99)a2-F	AACTCGAGATGGCCATCCAGCAGACCTCATTTG
XbaI-3A(O99)tC1-R	AATCTAGATTACTCGTTCACTGCATCATCC
NheI-3A-HindIII (-GFP or -GST)	
NheI-3A(O97)-F	AAAGCTAGCATGATTTCAATCCCTTCCCAGAAG
HindIII-3A(O97)-R	AAAAAGCTTTTCAGCTCGCGGTTGTTC
NheI-3A(O99)-F	AAAGCTAGCATGGCCATCTCAATTCCTTCCCAAAAGG
HindIII-3A(O99)-R	AAAAAGCTTTTCAGCTTGTGGTTGTTCTTC
HindIII-3A(O99)N-R	AAAAAGCTTGTTTTCCTTCAGGCGC
NheI-3A(O99)HR-F	AAAGCTAGCATGGCCTTTGAGATAGTTGCCCTGTG
HindIII-3A(O99)HR-R	AAAAAGCTTGATCATGATCACTATGTTTGCC
NheI-3A(O99)C-F	AAAGCTAGCATGGCCCGCGAGACTCGCAAG
HindIII-3A(O99)tC1-R	AAAAAGCTTCTCGTTCACTGCATCATCC
NheI-3A(O99)tC2-F	AAAGCTAGCATGGCCTACATTGAGAAGGCAAGCATC
HindIII-3A(O99)a1-R	AAAAAGCTTGAGAGGCCGGAGCTCC
NheI-3A(O99)a2-F	AAAGCTAGCATGGCCATCCAGCAGACCTCATTTG
Vectors or others	
NheI-GFP-F	AAAGCTAGCATGGGCAGCAAGGGCGAGG
XhoI-HindIII-GFP-R	AACTCGAGTCAAAGCTTCTTGTACAGCTCGTCCATG
NheI-BamHI-d1D-F	AAAGCTAGCGCCACCATGGGATCCCACAAGCAGAGGATTGTGG
NheI-BamHI-d1D2A-F	AAAGCTAGCATGCTCGAGCACAAGCAGAGGATTGTGG
XhoI-d1D2A-R	TTTCTCGAGGGGCCCAGGGTTGG

NheI-HindIII-GFP-F	AAAGCTAGCATGAAGCTTATGAGCAAGGGCGAGG
BamHI-GFP-R	AAAGGATCCCTTGTACAGCTCGTCCATG
NheI-GST-F	AAAGCTAGCATGGCCTCCCCTATACTAGGTTATTG
BamHI-HindIII-GST-R	AAGGATCCTTAAAGCTTTTTTGGAGGATGGTCGC
HindIII-GST-F	AAAAAGCTTATGGCCTCCCCTATACTAGGTTATTG
XhoI-GST-R	AACTCGAGTTATTTTGGAGGATGGTCGC
NheI-CALR-mCherry-F	AAAGCTAGCATGGCCCTGCTATCCGTGCCGCTGCTGCTCGGCCTCCTCGGCCTGGCCGTCGCCGAGATGGTGAGCAAGGGC
HindIII-KDEL-mCherry-R	AAAAAGCTTTTACAGCTCGTCCTTCTTGTACAGCTCGTCC
BamHI-sSar1-F	TTGGATCCATGTCTTTCATCTTTGAGTGGATCTAC
2B or 2C	
NheI-FLAG-2B-F	AAAGCTAGCATGGATTACAAGGATGACGACGATAAGGGTACCCCCTTCTTCTTCTCCGAC
EcoRI-stop-BamHI-2B-R	AAAGAATTCTTAGGATCCCTGCTTTTCTGCTCTCTCG
NheI-FLAG-KpnI-2C-F	AAAGCTAGCATGGATTACAAGGATGACGACGATAAGGGTACCCTCAAAGCACGTGACATCAAC
EcoRI-stop-BamHI-2C-R	AAAGAATTCTTAGGATCCCTGTTTAAATATCAGGTGGCTCG
Mutagenesis	
O973A-dNheI-F	GGAGACGAGTGGCGCCAGCGCTGTCGGTTTC
O973A-dNheI-R	GAAACCGACAGCGCTGGCGCCACTCGTCTCC
msSar1-H79G-F	TTTGATCTCGGTGGGGGTGAGCAAGCACGTCG
msSar1-H79G-R	CGACGTGCTTGCTCACCCCCACCGAGATCAAA
msSar1-T39N-F	ACAATGCAGGCAAAAACACTCTTTTACACAT
msSar1-T39N-R	ATGTGTAAAAGAGTGTTTTTGCCTGCATTGT
msSar1-QTTG-F	TTTGGGCTTTATGGAGCCGCCGCAGCAAAGGGGAATGTGACC
msSar1-QTTG-R	GGTCACATTCCCCTTTGCTGCGGCGGCTCCATAAAGCCCAAA
XhoI-msSar1-D198A-R	AACTCGAGTCAGGCAATATACTGGGAGAGCCAGC
sSec12-I41A-F	GCTGCCAAGACCGGTGCAAAGAACGGCGTGCAC
sSec12-I41A-R	GTGCACGCCGTTCTTTGCACCGGTCTTGGCAGC
m3A-49,50-F	CAGACCTCATTTGTGGCCGCCGCTTTTAAGCGCCT
m3A-49,50-R	AGGCGCTTAAAAGCGGCGGCCACAAATGAGGTCTG
m3A-53,54-F	GTGAAGCGCGCTTTTGCCGCCCTGAAGGAAAACTT
m3A-53,54-R	AAGTTTTCCTTCAGGGCGGCAAAAGCGCGCTTCAC
m3A-54,56-F	AAGCGCGCTTTTAAGGCCCTGGCGGAAAACTTTGAGAT
m3A-54,56-R	ATCTCAAAGTTTTCCGCCAGGGCCTTAAAAGCGCGCTT
m3A-80,81,82-F	CATGATCCGCGAGACTGCCGCCGCACAGCAGATGGTGGATG
m3A-80,81,82-R	CATCCACCATCTGCTGTGCGGCGGCAGTCTCGCGGATCATG
m3A-125,126,127-F	CACCACTGTTGGTTTCGCCGCCGCAACTCTCCCGGGACAC
m3A-125,126,127-R	GTGTCCCGGGAGAGTTGCGGCGGCGAAACCAACAGTGGTG

Green: restriction site; Red: start codon or stop codon; Blue: tag or additional signal peptide; Yellow: mutation site
